# Continuous variation in herkogamy enhances the reproductive response of *Lonicera implexa* to spatial variation in pollinator assemblages

**DOI:** 10.1093/aobpla/plz078

**Published:** 2019-12-05

**Authors:** Amparo Lázaro, Jaume Seguí, Luis Santamaría

**Affiliations:** 1 Global Change Research Group, Mediterranean Institute for Advanced Studies (IMEDEA; UIB-CSIC), Esporles, Balearic Islands, Spain; 2 Doñana Biological Station (EBD-CSIC), Sevilla, Spain

**Keywords:** Corolla length, fruit set, *Macroglossum stellatarum*, pollinator visitation, seed germination, seed set, seed weight, selfing capability, stigma-anthers distance

## Abstract

Herkogamy, the spatial separation of sex organs in hermaphroditic plants, has been proposed as a mechanism to reduce self-pollination and the associated processes of inbreeding and gamete wastage. Longitudinal herkogamy is the most frequent type, with two subtypes: approach herkogamy (anthers below the stigma), which is associated with diverse pollinator arrays, and reverse herkogamy (anthers above the stigma), associated with specialized, long-tongued pollinators. By using a herkogamy index that varied continuously from negative (reverse herkogamy) to positive (approach herkogamy) values, we studied the effect of continuous variation in herkogamy on pollinator attraction, selfing capability and plant fitness across three populations of *Lonicera implexa* differing in the relative abundance of long-tongued vs. short-tongued pollinators. Reverse herkogamy was significantly more frequent in the population where long-tongued pollinators were dominant than in the other two populations. Agreeing with this, the main floral visitors of *L. implexa* individuals with small and large herkogamy index were, respectively, long-tongued and short-tongued pollinators. Spontaneous selfing was low and increased with increasing herkogamy index (i.e. with approach herkogamy), although most of it occurred when there was close distance between anthers and stigma. Fruit production was unrelated to the herkogamy index in the population with long-tongued pollinators, but it increased with approach herkogamy (higher herkogamy index) in the other two populations. In contrast, seeds of individuals with reverse herkogamy (smaller herkogamy indices) germinated better. In this species, continuous variation in herkogamy might function as a reproductive strategy, as different morphotypes might be favoured by different pollinator assemblages.

## Teaser

By using a herkogamy index that varied continuously from negative (reverse herkogamy) to positive (approach herkogamy) values, this study evaluates the effect of continuous variation in herkogamy on pollinator attraction, selfing capability and plant fitness in *Lonicera implexa* populations differing in the relative abundance of long-tongued vs. short-tongued pollinators. The results show that different morphotypes might be favoured by different pollinator assemblages, and that continuous variation in herkogamy in this plant species might function as a reproductive strategy, optimizing seed quality when long-tongued pollinators are abundant, and increasing fruit set when they are rare.

## Introduction

Herkogamy is the spatial separation of sex organs in hermaphroditic plants and it is thought to have evolved in plants with different pollination modes to reduce self-pollination and to limit gamete wastage (Webb and Lloyd 1986; Barrett 2002a, b, 2003). However, empirical evidence to support these hypotheses is still very scarce. In approach herkogamy, the stigma is presented above the anthers, whereas in reverse herkogamy, the anthers are located above a recessed stigma (Barrett 2003). In animal-pollinated plants, this means that pollinators contact the stigma first when visiting a flower with approach herkogamy, whereas they contact the anthers first when visiting one with reverse herkogamy (Barrett 2003). In animal-pollinated plants, approach herkogamy is the most prevalent type of herkogamy and it is associated with a broad variety of pollinators. Reverse herkogamy is less common and it is supposed to be related to long-tongued pollinators, such as lepidoptera, when accompanied by long and narrow corolla tubes (Webb and Lloyd 1986; Barrett 2003), although not all plant species pollinated by lepidoptera display reverse herkogamy (Murcia 1990; Parra-Tabla and Bullock 2005).

While some plant species are polymorphic (i.e. they display two or several, discrete herkogamy morphs), most species are monomorphic, showing no discrete morphs and different extents of continuous variation in herkogamy. The majority of studies on herkogamy are focused on one type of polymorphism, the heterostyly, a reciprocal form of herkogamy in which there are two or three morphs that differ reciprocally from one another in the same breeding population (Barrett *et al.* 2000; Barrett 2002b), as well as on other non-reciprocal polymorphisms related to heterostyly, but in different stages in their evolution (e.g. Pérez-Barrales and Arroyo 2010; Pérez-Barrales *et al.* 2018). The study of continuous variation in herkogamy in monomorphic species has received lesser attention, and has been mostly studied in relation to its effect on self-pollination (e.g. Parra-Tabla and Bullock 2005; Fishman and Willis 2007; de Vos *et al.* 2012) and to the developmental changes during anthesis that occur in some species (Barrett 2002a; Armbruster *et al.* 2009; de Vos *et al.* 2012). However, some other plant species exhibit continuous variation in the separation of sex organs from reverse to approach herkogamy that do not change with floral development (Kulbaba and Worley 2008, 2012, 2013; Forrest *et al.* 2011). These continuous variation deserves further attention, because the positions of sex organs can have profound implications for the mating biology of populations (Barrett 2002b), but also because a continuum including both approach and reverse herkogamy cannot be explained simply as a mechanism to reduce self-pollination (Kulbaba and Worley 2008). Instead, this variation could reflect contrasting selection by dissimilar pollinators. For instance, in *Polemonium brandegei*, a species pollinated by both hawkmoths and hummingbirds and that exhibit a continuous variation from reverse to approach herkogamy, it has been shown that hawkmoths select for reverse herkogamy, whereas hummingbirds select for approach herkogamy (Kulbaba and Worley 2012, 2013). In this plant species, hawkmoth selection for reverse herkogamy seems to be mediated both by increased pollinator efficiency due to higher flower-pollinator match, and by increased pollinator attraction to this type of herkogamy, hypothesized to be mediated by lower obstruction of the opening of corolla tubes when fewer sex organs are exerted (Kulbaba and Worley 2012). On the other hand, selection may sometimes favour reduced herkogamy if a close proximity of stigma and anthers is necessary to ensure that small pollinators contact both sex organs (Sahli and Conner 2011), and to achieve spontaneous self-pollination when pollinators are scarce (de Vos *et al*. 2012). However, very little is still known about the maintenance of these variations in the type and degree of herkogamy.

In this study, we document the continuous variation in herkogamy in *Lonicera implexa*, which goes from strong reverse herkogamy to strong approach herkogamy. Then, we assess whether this variation was simply a consequence of the variation in corolla length that the species exhibit (Lázaro *et al.* 2015; Lázaro and Santamaría 2016), since in this species the stamen’s filament is short and fused to the inner part of a corolla tube but the stigma is free (Devesa and Ruiz 2007). In addition, we evaluate the relationship between the degree and type of herkogamy and the visitation of long- vs. short-tongued pollinators, the capability of self-pollination and plant fitness in three populations differing in the importance of the diurnal hawkmoth, *Macroglossum stellatarum*, as pollinator. Our main hypothesis was that herkogamy diversity in this species may be maintained as a bet-hedging strategy that maximizes reproductive output across the range of variation in its pollinator environment, so that differences among sites in pollinator assemblages are related to differences in the degree and type of herkogamy. Specifically, we aimed to test whether: (i) the continuous variation in herkogamy in this species correlated with corolla dimensions; (ii) long-tongued pollinators preferentially visit *L. implexa* flowers displaying reverse herkogamy; (iii) spontaneous selfing increases as the distance between anthers and stigma was reduced; and (iv) approach herkogamy is favoured in populations with short-tongued pollinators, whereas reverse herkogamy was favoured when long-tongued pollinators (mainly hawkmoths) were abundant.

## Materials and Methods

### Study species

The study species was the Mediterranean honeysuckle, *L. implexa*, a climbing shrub that occurs in Southern Europe, mainly in the Mediterranean coast and south and central Spain. It flowers from late April to June, and its inflorescences bear approximately six hermaphroditic and zygomorphic flowers whose colour varies from pink (before opening) to white-yellowish (once open). Flowers have long corolla tubes that vary considerably in length, both within and among individual plants (ca. 14–47 mm, Devesa and Ruíz 2007; Lázaro *et al.* 2015; Lázaro and Santamaría 2016). In *L. implexa*, as in many other species with tubular flowers, the stamen’s filament is short (0–4.1 mm in Devesa and Ruíz 2007) and its base is fused to the inner part of a corolla tube, while the stigma is free and long, departing from a basal disk, and shows a broad range of variation (Devesa and Ruíz 2007). *Lonicera implexa* suffers nectar robbing and it is pollinated by longue-tongued pollinators such as the hummingbird hawkmoth (*M. stellatarum*) and butterflies, but also by other insects with shorter tongues, such as bees, flies and beetles; and the importance of the main pollinator differs among populations (Lázaro and Santamaría 2016). To our knowledge, there is no previous published information about the breeding system of this species. Each flower has 3–4 carpels that develop, when fecundated, into fleshy orange fruits (drupes) that are mature by the end of August.

### Study populations

We conducted our study in three populations of *L. implexa* separated by ca. 4 km from each other (range of distances: 3.3–4.5 km) and located in a mosaic of oak-pine forests and garrigue shrubland at the Tramuntana Mountains in Mallorca (Balearic Islands, Spain). The three populations were: (i) Establiments (39°38′54.56″N, 2°36′25.84″), at ca. 160 m altitude; (ii) Son Tries (39°39′38.19″N, 2°34′32.66″E), at ca. 260 m altitude; and (iii) Banyalbufar (39°40′55.70″N, 2°31′47.06″E), at ca. 360 m altitude. While Establiments and Son Tries were located on southern slopes, Banyalbufar was on a northern slope. There was an increased importance of *M. stellatarum* as pollinator with increasing altitude in these populations (Lázaro and Santamaría 2016). While in Bayalbufar the great majority of flower visits were conducted by hawkmoths, in Son Tries both social bees and hawkmoths were equally important as pollinators and in Establiments hawkmoths were virtually absent and flower visitation was mainly conducted by small beetles (Lázaro and Santamaría 2016). Corolla tube length is a key trait in these populations since it is related to reward accessibility in the flowers and plant fitness (Lázaro *et al.* 2015; Lázaro and Santamaría 2016). In the two study populations where short-tongued insects are important pollinators (Son Tries and Establiments) short corollas are selected by pollinators (see accessibility index, which increased with the decrease of corolla length and the increase in corolla width; Lázaro and Santamaría 2016).

### Flower measurements

In the spring 2012, we measured in the field the distance between the stigma and the anthers by using a digital calliper, and noted the relative position of the sex organs, i.e. whether the stigma was above (approach herkogamy) or below the anthers (reverse herkogamy). Measurements were conducted in 20 flowers belonging to five inflorescences (four per inflorescence) in all individuals that flowered in the study populations (33, 38 and 38 in Banyalbufar, Establiments and Son Tries, respectively). These flowers were selected to be in the same developmental stage, i.e. completely open and receptive. In these same flowers we also measured the length and width of the corolla tube.

Using these data, we calculated a herkogamy index for each flower as a continuous variable that measures the distance between the stigma and the anthers and goes from negative values when the stigma is below the anthers (reverse herkogamy) to positive values, when the stigma is above the anthers (approach herkogamy).

### Pollinator visitation

We performed a total of 312 pollinator censuses of 15 min, from 4 May to 4 June 2012 at the three populations, covering the complete flowering period of these three populations the study year. The observations were made separately on each focal, individually marked plant (Banyalbufar: 27; Establiments: 36; Son Tríes: 33), and conducted every day from 0900 to 2130 h, unless windy or cloudy/rainy conditions curtailed pollinator activity. Each day we observed all the three populations in different order, so all the populations were observed during all the day periods. We aimed at observing every flowering individual at least during three observation periods, including the different periods of the day (average number of censuses conducted per individual: 3.74 ± 0.41, 3.03 ± 0.19 and 3.00 ± 0.16, for Banyalbufar, Establiments and Son Tries, respectively). The average number of flowers observed per individual plant in an observation period was 84.0 ± 30.9, 102 ± 18.1 and 86.1 ± 12.4, for Banyalbufar, Establiments and Son Tries, respectively. We complemented these diurnal censuses with 18 nocturnal censuses of 15 min at each population, conducted from 2130 to 2330 h, using a red light to reduce pollinator disturbance. None of these censuses found nocturnal moths pollinating the species; therefore, we assumed that nocturnal pollination might be of limited relevance in our study system (as reported for other *Lonicera* species, Guitián *et al.* 1993) and will report only on the results of the diurnal censuses.

During each observation period we noted the number of insect visits to the focal plant. A pollinator visit was defined to have occurred when there was contact between the visitor’s body and the reproductive organs (stigma or anther) of the flower. We categorized the flower visitors into long-tongued species (hawkmoths with 24–27 mm of proboscis length; Zlatkov *et al.* 2017, and butterflies, e.g. *Cynthia cardui* and *Colias crocea*, with proboscides of 14–15 mm; Corbet 2000) and short-tongued species (social and solitary bees, small beetles, hoverflies and muscoid flies; << 10 mm proboscis length; authors’ unpublished data). After each observation period, we estimated the total number of open flowers of each individual plant, and used these data to estimate the total number of visits by both long- and short-tongued pollinators per plant. To evaluate differential preference by each of these groups of flower visitors, we calculated the proportion of long-pollinators respect to the total pollinators visiting each individual plant during each observation census.

### Capability of spontaneous self-pollination

In spring 2012, to estimate spontaneous selfing, we bagged two closed inflorescences in 18, 31 and 30 individuals in Banyalbufar, Establiments and Son Tries, respectively. In these same individuals, we marked other two inflorescences that were left open to natural pollination (controls). All the flowers of these inflorescences were counted (23.2 ± 2.1 and 24.9 ± 1.8 flowers per study individual in bagged and control inflorescences, respectively). At the beginning of July, when fruits started to initiate, we removed the bags to allow a correct development of the fruits. In September, we counted the fully developed fruits produced by these inflorescences. For each individual we obtained an autogamy index (AI) estimated as: AI = (FS_selfing_ − FS_open-pollination_)/FS_maximum_, where FS_selfing_ is the fruit set (fruits/flower) obtained from the bagging treatment, FS_open-pollination_ is the fruit set obtained from the open-pollination treatment in the same individual, and FS_maximum_ = FS_selfing_ when FS_selfing_ > FS_open-pollination_, and FS_maximum_ = FS_open-pollination_ when FS_open-pollination_ > FS_selfing_. The index ranges from −1 to 1, in an analogous manner as the ‘Relative performance of crosstypes (RP)’ index by Ågren and Schemske (1993). Positive values in our index indicate that spontaneous selfing outperforms open-pollination, and negative values of the index indicate that open-pollination outperforms selfing. Although in our experiment the inflorescences open to natural pollination could also self-pollinate, lower fruit set in open-pollination as compared to selfed flowers could occur if inefficient pollinators decrease both outcrossing and selfing success. Selfing success could be reduced in the open-pollination treatment compared to the selfing treatment if there was: (i) efficient pollen removal from anthers, which deplete anthers’ pollen before self-pollination occurs; and/or (ii) stigma clogging with pollen from other species (or incompatible genotypes), which blocks stigma receptivity before selfing takes place. As final fruit production was zero in many inflorescences (similarly in all the populations), to calculate the indices for each individual plant we merged the data of the two inflorescences of each treatment per individual.

### Plant fecundity

In spring 2012, to estimate plant fecundity, we haphazardly selected and marked five inflorescences in each study individual (Banyalbufar: 33; Establiments: 36; Son Tríes: 33) and counted the total number of flowers in those inflorescences. In September, when fruits were mature, we counted the number of fruits produced in these inflorescences and collected them for dissection in the laboratory, where the number of fully developed seeds in them was counted and each seed individually weighted (±1 mg). Weighted seeds were individually sown in labelled plugs within watered germination trays at IMEDEA’s greenhouse. Seed germination was followed every third day for over 2 months, until no new germinations occurred. We used fruit set (fruits/flower), seeds/fruit, seed weight and seed germination as measures of plant fecundity.

### Statistical analyses

All the analyses were conducted in R ver. 3.4.3 (R Core Team 2017). We used a general linear mixed model (package lme4, function lmer; Bates *et al.* 2015) to assess how the herkogamy index varied among populations and with corolla tube length and width. The full model included population, corolla length and width and their interactions, because previous Variance Inflation Factor (VIF) analyses showed no collinearity between these predictor variables (all VIF values < 2; Zuur *et al.* 2009). The model included also individual and inflorescence nested within individual as random factors.

We conducted separate analyses to test the relationship between the herkogamy index and the attraction to long-tongued pollinators, the selfing capability (AI) and each fecundity variable (fruit set, seeds/fruit, seed weight and seed germination). We used generalized linear models (package nlme; Pinheiro *et al.* 2018) for the models of selfing capability and fruit set, as individual plants were the sampling units; in these models we used the average herkogamy index to characterize each individual. For the other response variables, we used generalized linear mixed models (package lme4; Bates *et al.* 2015) with individual plant as random factor in the models of proportion of visits by long-tongued pollinators and seeds/fruit; and plant, inflorescence and fruit as random factors in the models of seed weight and germination. Due to the nature of the data, we used: (i) binomial error distributions and logit link functions for the analyses of proportion of visits by long-tongued pollinators, fruit set and seed germination; (ii) a gamma error distribution with log as a link function for the model of selfing capability; (iii) a poisson error distribution and log link function for the analysis of seeds/fruit, after checking for the absence of overdispersion (Zuur *et al.* 2009); and (iv) a gaussian error distribution and identity link function for the analysis of seed weight. Full models included the herkogamy index as a continuous predictor variable, population as a fixed factor, and the interaction between population and the herkogamy index. Seed mass was added as an additional covariable in the model of seed germination. In all the cases, we used automatic model selection based on AIC (function *dredge*, package MuMIn; Barton 2018) to find the best models among the set of combinations of predictor variables and their interactions, setting the herkogamy index as a variable that should be always included in the models, since it was the main variable that we were interested to test. Significance of variables is based on likelihood ratio tests (LRT). After each model, we ran Tukey *post hoc* tests to determine significant differences between levels of each significant categorical variable or interaction (function *lsmeans*, package lsmeans; Lenth 2016). Only best models are reported in the Results section, where predicted means and model estimates are accompanied by their standard error.

## Results

### Variation in the herkogamy index and its relationship with corolla length and width

**Figure 1.**
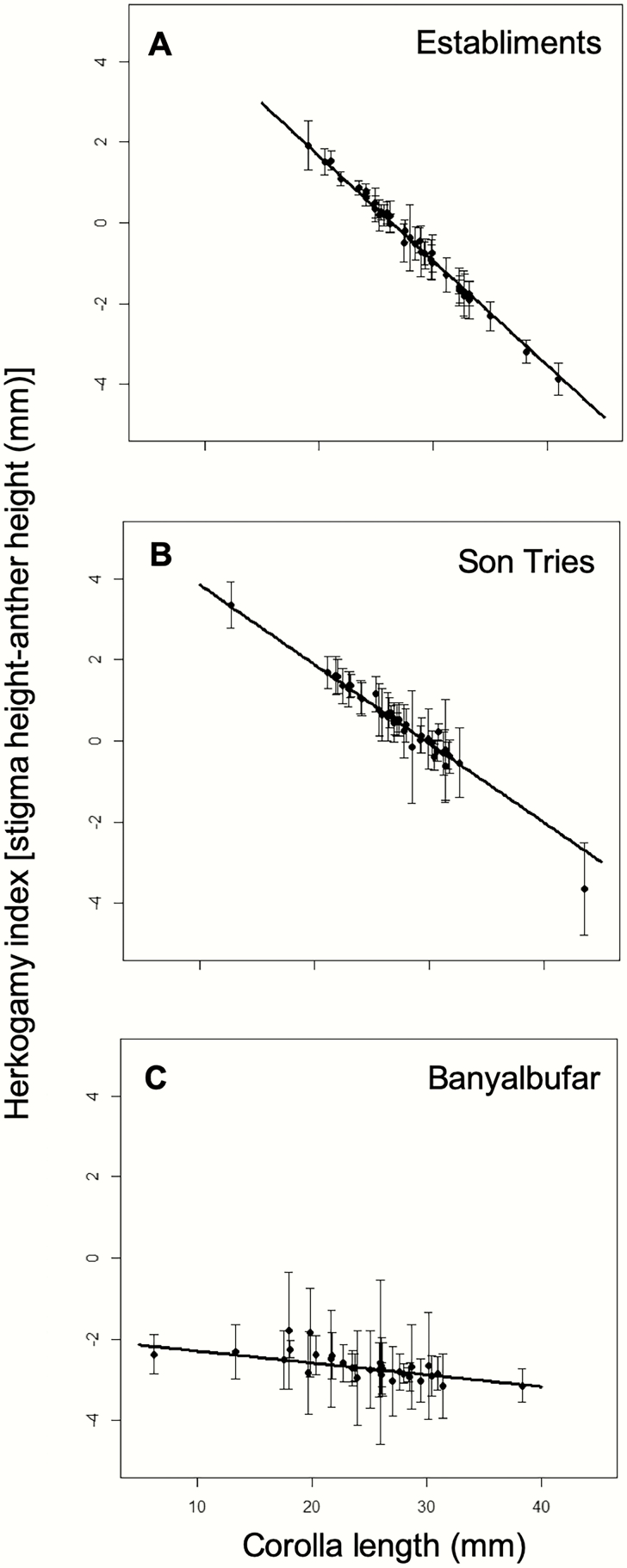
Relationship between the herkogamy index and corolla length in each study population. The average (±SE) partial residuals of the model for each individual plant are shown. Lines represent the estimates for the best model in each population. Establiments (A), Son Tries (B), Banyalbufar (C). Interaction herkogamy index × population: *P* < 0.0001.

Herkogamy indices varied from −22.33 (reverse herkogamy) to 27.70 mm (approach herkogamy). Overall, the individuals from Banyalbufar showed significantly smaller herkogamy indices (i.e. higher reverse herkogamy) than those from Establiments and Son Tries (−3.16 ± 0.18, −0.40 ± 0.14 and 0.66 ± 0.14, for Banyalbufar, Establiments and Son Tries, respectively; χ ^2^ = 96.54, df = 2, *P* < 0.0001). The relationship between corolla length and the herkogamy index differed also among populations (corolla length × population: χ ^2^ = 80.88, df = 2, *P* < 0.0001). In Establiments and Son Tries the herkogamy index decreased significantly with corolla length (Fig. 1A and B), whereas in Banyalbufar, the herkogamy index was not related to corolla length (Fig. 1C).

**Figure 2.**
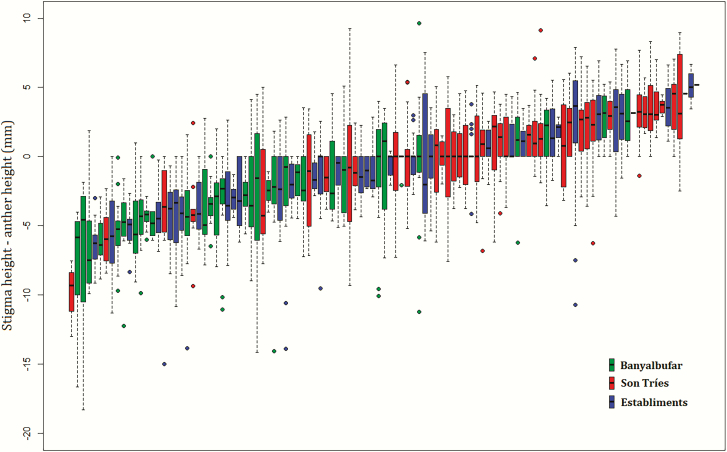
Boxplots showing the herkogamy index for each study individual. Red: individuals from Banyalbufar; blue: individuals from Establiments; green: individuals from Son Tries.

Although there was considerable variation among and within individuals in each study site (Fig. 2), the populations differed clearly in the dominant morphotype: in Banyalbufar, most individuals showed reverse herkogamy; in Son Tríes, most individuals showed approach herkogamy; while in Establiments, a fairly homogeneous proportion of individuals appeared across the whole range of values of the herkogamy index (Fig. 2).

### Relationship between the herkogamy index and pollinator visitation

**Figure 3.**
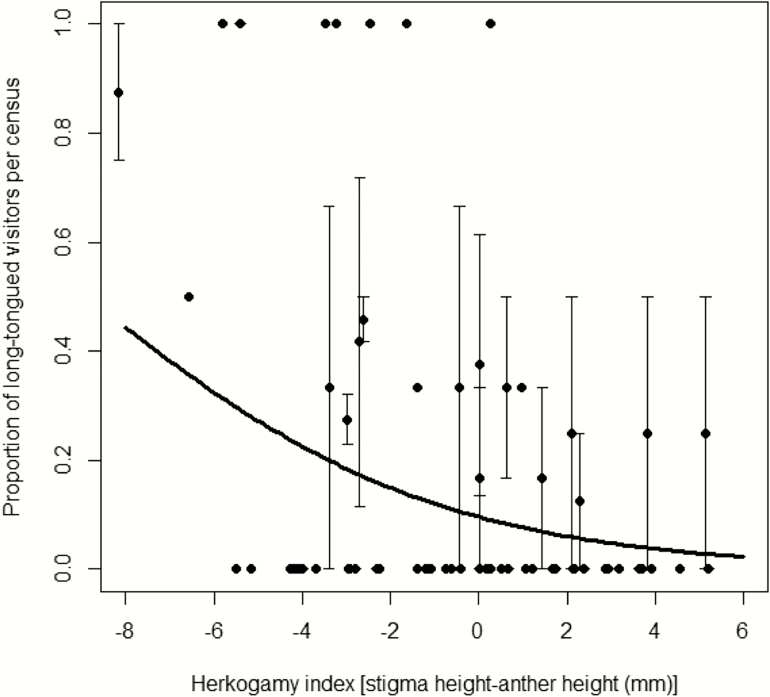
Relationship between the herkogamy index and the proportion of long-tongued visitors per observation census. Line represents the estimate for the best model and the dots represent the average (±SE) of the proportion of long-tongued visitors per observation census on an individual plant. Herkogamy index: *P* = 0.007.

Reverse herkogamy was associated with more visits by long-tongued pollinators (i.e. decreasing the herkogamy index resulted in a considerable increase in the ratio of long-tongued vs. short-tongued pollinator visits) across the three populations (Table 1A; Fig. 3). Visits by long-tongued pollinators were more frequent in Banyalbufar than in the other two populations, although *post hoc* analyses showed that the differences were only significant between Banyalbufar and Establiments (0.48 ± 0.08, 0.22 ± 0.05 and 0.02 ± 0.01, for Banyalbufar, Son Tries and Establiments, respectively; Table 1A). Visitation recorded by different pollinator groups is given in **Supporting Information—Table S1**.

**Table 1.** Results of the best models showing the relationship between the herkogamy index and insect visitation, capability of spontaneous selfing and fecundity variables. For each variable that appear in the best models, the χ ^2^, the degrees of freedom (df) and the *P*-value are shown. The variables involved in significant interactions were also included in the full model. Significant values are marked in bold. The herkogamy index was fixed while selecting the best models, because we were specifically interested in testing its effect on the response variables.

Model	Variable	χ ^2^	df	*P*
A) Proportion of visits of long-tongued pollinators	Herkogamy index	7.33	1	**0.007**
	Population	22.29	2	**<0.0001**
B) Autogamy index	Herkogamy index	5.07	1	**0.024**
C) Fruit set	Herkogamy index × Population	32.20	2	**<0.0001**
	Population	56.42	2	**<0.0001**
D) Seeds per fruit	Herkogamy index	1.10	1	0.294
	Population	16.59	1	**0.0003**
E) Seed weight	Herkogamy index	1.08	1	0.299
	Population	9.35	1	**0.009**
F) Seed germination	Herkogamy index	6.36	1	**0.012**
	Seed weight	27.57	1	**<0.0001**

### Relationship between the herkogamy index and selfing capability

**Figure 4.**
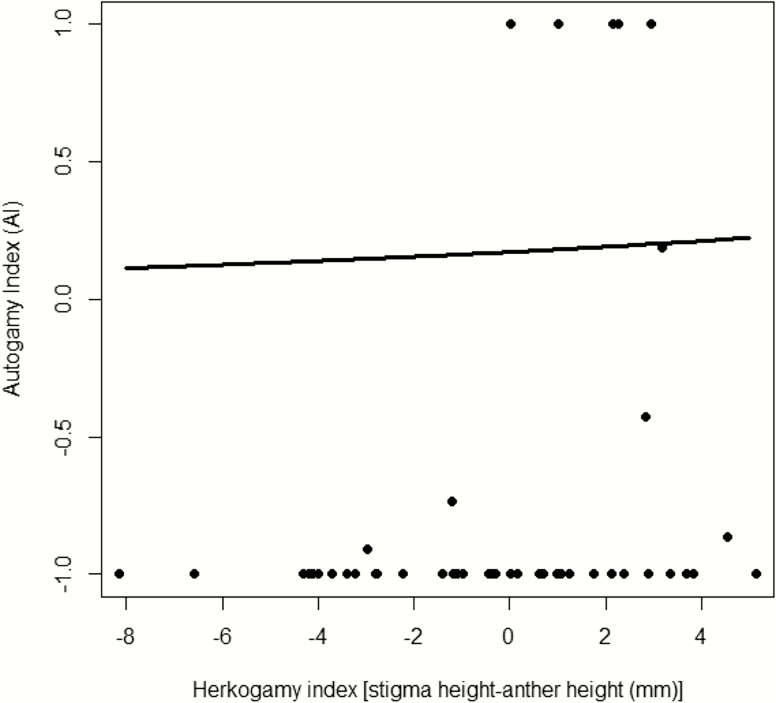
Relationship between the herkogamy index and the autogamy index (AI = (FS_selfing_ − FS_open-pollination_)/FS_maximum_, where FS is fruit set). Line represents the estimate for the best model and the dots represent the AI for each study plant. Herkogamy index: *P* = 0.024.

Spontaneous selfing events were few. They occurred only in 10 out of 79 bagged individuals and, in these 10 plants, the average number of selfed fruits was 1.8 ± 0.33. Selfing events took place at intermediate herkogamy values, i.e. when the separation between anthers and stigma was small (−4 to 4 mm; Fig. 4). The AI could be calculated only for 45 individual plants, as the rest had no fruits either in the bagging or in control branches and were then excluded from the analysis. These individuals did not differ in herkogamy from those used to calculate AI (range: −6.8 to 5.2 vs. −6.6 to 5.1, respectively; χ ^2^ = 1.74, df = 1, *P* = 0.186). Considering these 45 individuals, we only obtained 18 selfed fruits out of the 1044 flowers bagged (1.7 %), while fruit set in the controls was 17 % (190/1119). The AI increased significantly with the herkogamy index (Table 1B), indicating that more spontaneous selfing occurred in individuals with increased approach herkogamy (Fig. 4).

### Plant reproductive success

**Figure 5.**
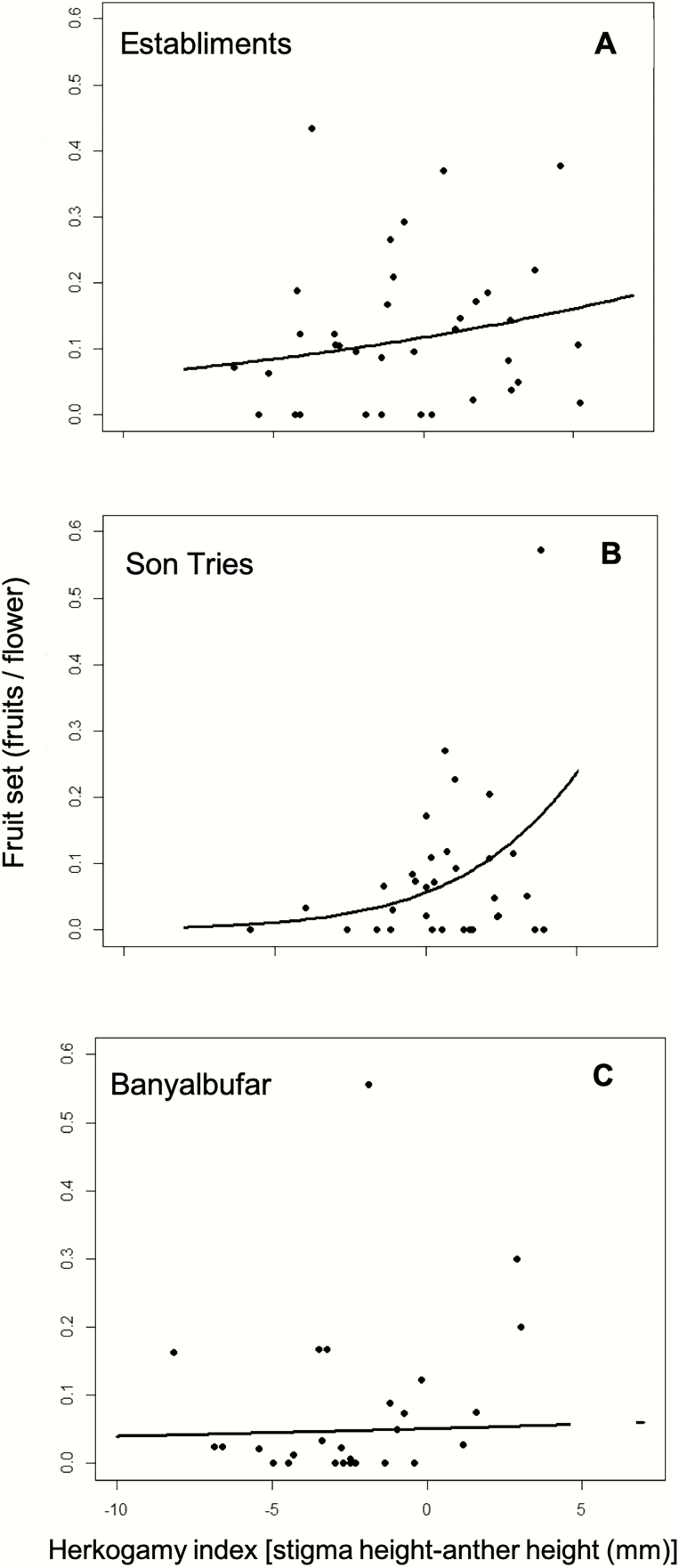
Relationships between the herkogamy index and fruit set in each study population. Lines represent the estimates for the best model in each population and the dots represent the values of fruit set for each individual study plant. Establiments (A), Son Tries (B), Banyalbufar (C). Interaction herkogamy index × population: *P* < 0.0001.

The herkogamy index had significant effects on both fruit set and seed germination. However, the effect of herkogamy on fruit set varied among populations (significant herkogamy index × population interaction; Table 1C). In Son Tries and Establiments, fruit set increased with the herkogamy index (i.e. higher approach herkogamy; Fig. 5A and B), while in Banyalbufar there was no relationship between the herkogamy index and fruit set (Fig. 5C). Populations also differed in overall fruit set, with Establiments having overall higher fruit set than the others (0.13 ± 0.029 vs. 0.08 ± 0.02 and 0.08 ± 0.02, for Establiments, Banyalbufar and Son Tries, respectively).

The number of seeds per fruit and seed weight were not significantly related to the herkogamy index (Table 1D and E). However, both variables differed significantly among populations (Table 1D and E). The fruits from Banyalbufar had significantly fewer seeds than those from Establiments and Son Tries (0.33 ± 0.08 vs. 1.43 ± 0.07 and 1.49 ± 0.1, respectively), and also weighted less (0.006 ± 0.0007 vs. 0.010 ± 0.0002 and 0.010 ± 0.0002 mg, for Banyalbufar, Establiments and Son Tries, respectively).

**Figure 6.**
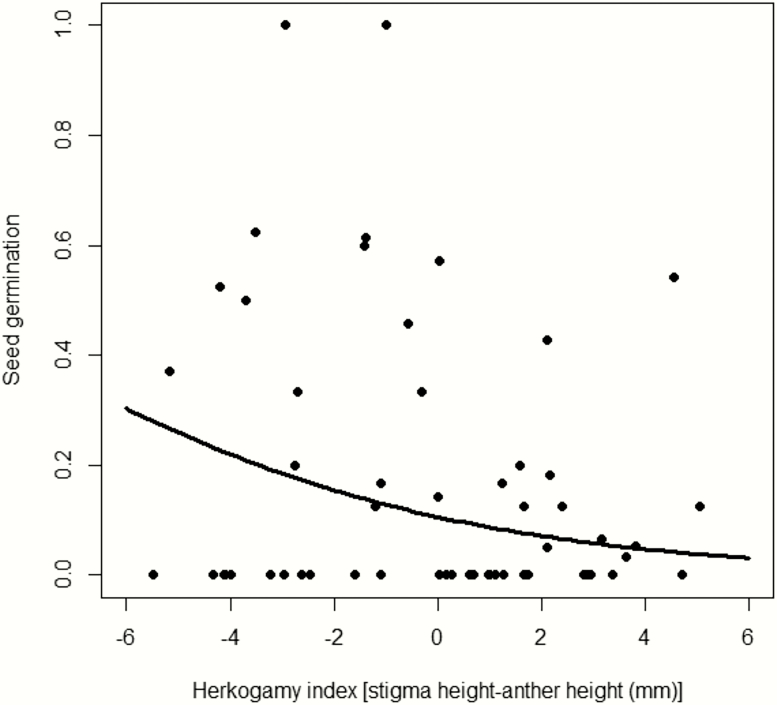
Relationships between the herkogamy index and seed germination. Line represents the estimate for the best model and the dots represent the proportion of germinated seeds for each individual plant. Herkogamy index: *P* = 0.012.

Seed germination (Table 1F; Fig. 6) decreased significantly as the herkogamy index increased, indicating that plants with flowers showing reverse herkogamy sired seeds that germinated better. Seed weight had an additional positive effect on germination (Table 1F).

## Discussion

*Lonicera implexa* showed a large variation in the degree and type of herkogamy. As hypothesized, reverse herkogamy was more frequent in the population where the flower-visitor community was dominated by long-tongued pollinators, as compared to those with short-tongued pollinators. Likewise, long-tongued pollinators visited preferentially plants with reverse herkogamy, while short-tongued pollinators preferred plants displaying approach herkogamy across the three populations. Spontaneous selfing was low and, contrary to our expectations, increased with the herkogamy index, although most of it occurred when the distance between anthers and stigmas was very low. Fruit production was unrelated to herkogamy in the population where long-tongued pollinators dominated, while it increased with approach herkogamy in the other two populations. In contrast, individuals with reverse herkogamy sired seeds that germinated better. These results suggest that, in this system, continuous variation in herkogamy is maintained because the different morphotypes are favoured locally by the contrasting pollinator assemblages.

### Continuous variation in herkogamy in *Lonicera implexa*

In monomorphic species, continuous variation in herkogamy values (distance between stigma and anthers) is not uncommon (e.g. Parra-Tabla and Bullock 2005; Fishman and Willis 2007; De Vos *et al.* 2012). Yet continuous variation spanning from reverse to approach herkogamy, as we found for *L. implexa*, has only been reported in a few species and with much smaller ranges of herkogamy values (Kulbaba and Worley 2008; Forrest *et al.* 2011).

In species as *L. implexa*, with individuals that differ considerably in corolla length (Lázaro *et al.* 2015; Lázaro and Santamaría 2016), variation in herkogamy may be dependent on variation in corolla length—e.g. if anthers change their position, relative to the stigma, due to changes in the corolla tube length. However, we have shown here that the relationship between herkogamy and corolla tube length varied among populations. Indeed, *L. implexa*’s herkogamy index decreased with corolla length in the two populations with short-tongued pollinators, while it was independent of corolla length in the population with long-tongued ones, suggesting that reverse herkogamy, which predominates among the latter population, is a trait directly selected by such long-tongued pollinators, independently of corolla length. The interrelated effects of long-tongued pollinators on both herkogamy and corolla length may be triggered by two complementary mechanisms: selection for reverse herkogamy due to positional effects (Kulbaba and Worley 2012) and selection for long corolla tubes due to resource partitioning effects (Santamaría and Rodríguez-Gironés 2015; see Fig. 3 of Rodríguez-Gironés and Santamaría 2004 for a graphical summary of the process). Under such scenario, long corollas would attract long-tongued pollinators in populations where they are not dominant, triggering selection for reverse herkogamy in plants with long corollas (and thus an association between corolla length and herkogamy); while in assemblages dominated by long-tongued pollinators, they will visit all flowers—so that selection for reverse herkogamy would operate on all plants, independent of corolla length. Indeed, previous work on these populations supports this hypothesis, as in Banyalbufar (the population where hawkmoths dominate), display traits have a stronger relationship to *M. stellatarum* visits than corolla tube length; while in Son Tries (the mixed population), corolla tube length and width are the determinant traits affecting the identity of pollinator visits (Lázaro and Santamaría 2016).

### Variation in herkogamy and its effects on self-pollination

Herkogamy is hypothesized to evolve as a mechanism that reduces the negative effects of self-pollination: inbreeding depression in compatible species and gamete wastage in incompatible ones (Webb and Lloyd 1986; Barrett 2002a, b, 2003). Indeed, most studies show that self-pollination increases as herkogamy (stigma-anther separation) decreases (Murcia 1990; Parra-Tabla and Bullock 2005; Fishman and Willis 2007). But decreasing self-pollination does not come free of risks: it results in failed reproduction whenever (or wherever) plant and/or pollinator abundances are low. In such cases, developmental reduction of herkogamy (i.e. a reduction in herkogamy through floral development) can make self-pollination unlikely at the beginning but increasingly likely at the end of floral development—thus enhancing reproductive assurance, via delayed autonomous selfing (Armbruster *et al.* 2002; de Vos *et al.* 2012). Although during sampling we did not detect any indication of developmental changes in herkogamy in this species, we restricted our measurements to a specific developmental stage, and therefore, we cannot evaluate this possibility with our data.

In this study, we found that the levels of spontaneous selfing were very low and always around small values of separation between stigma and anthers. Surprisingly, we also show that the capability of spontaneous self-pollination increased with approach herkogamy in *L. implexa*. This result contradicts Webb and Lloyd’s (1986) and Barrett (2003) assertions that for most floral architectures, approach herkogamy functions more effectively than reverse herkogamy in preventing intra-floral self-pollination and enhancing outcrossing rates. It is worth noting, however, that flower position could influence the probability of autogamy occurring in an approach herkogamous flower. Although *L. implexa* flowers tend to be relatively erect or horizontal, rather than pendant, we did not collect data on flower position. Therefore, it is possible that changes in flower position affected the relationship between autogamy and approach herkogamy in this species. Alternatively, it could be that, in some cases, bagging provoked artefact contacts between stigmas and anthers (within or between flowers) or that the positive relationship between herkogamy index and self-pollination was a consequence of the differential effect of dichogamy in approach herkogamous flowers, not considered here (Cesaro *et al.* 2004). Besides, no selfing event was detected at stigma-anther distances larger than 4 mm; and, since these large values only occurred in the negative range of the herkogamy index for the individuals included in the experiment (Fig. 4), we cannot rule out that the correlation found is simply a consequence of an increase in selfing with decreasing stigma-anther distance (as reported by Murcia 1990; Parra-Tabla and Bullock 2005; Fishman and Willis 2007). Future studies might evaluate the role of intra-plant variation in the degree of herkogamy and selfing rates, as it might be that intra-plant variation in herkogamy helps to deal with the unpredictability of per-flower visitation rates by maintaining (low levels of) seed production in the absence of pollinator visits. In any case, our results suggest that *L. implexa*’s continuous variation in herkogamy (from approach to reverse herkogamy) cannot be explained simply as a mechanism to reduce self-pollination and increase outcrossing rates, similar to that reported by Medrano *et al.* (2012) for a species with approach herkogamy, *Narcissus longispathus*.

### Variation in herkogamy as a result of differences in pollinator environments

Our study populations differed considerably in the abundance of *M. stellatarum* (Lázaro and Santamaría 2016). These differences among populations are not likely due to an inability of *M. stellatarum* to fly across them, because this hawkmoth is a strong flier, able to cover very long distances during migration (Cánovas *et al.* 2015 and references therein), but rather by habitat preferences and/or space-use patterns. It is important to note, however, that this is an abrupt terrain where movement is relatively restricted; and that the three populations differ substantially in the orientation (thus probably in temperatures in early spring) and abruptness (from relatively flat terrain in Establiments to a strong slop in Banyalbufar). In addition, differences in pollinator distribution could reflect, at least partially, the observed differences in nectar accessibility (resulting from variations in corolla length and width; Lázaro and Santamaría 2016)—since *L. implexa* represents one of the most important nectar resources at these sites during that period of the year.

Among-population variation in herkogamy seemed well-fitted to the variation in pollinator assemblages. In the population where the hummingbird hawkmoth *M. stellatarum* was the main pollinator (Lázaro *et al.* 2015; Lázaro and Santamaría 2016), most *L. implexa* plants had flowers with reverse herkogamy (i.e. with lower herkogamy indices; Banyalbufar, Fig. 2). This is in agreement with the association between reverse herkogamy in flowers with long and narrow corolla tubes and pollination by longue-tongued lepidopterans (Webb and Lloyd 1986). Although the functional mechanisms behind this relationship are not totally clear (Barrett 2003), Kulbaba and Worley (2012) discussed increased potential for the narrow proboscis of hawkmoths to contact the sex organs within a narrow corolla tube. Alternatively, this association may be related to the fact that if stigmas are not deep enough within corolla tubes, they can be pollinated by short-tongued pollinators. In contrast, the populations where short-tongued pollinators were abundant (Lázaro *et al.* 2015; Lázaro and Santamaría 2016) individuals showed mostly approach herkogamy (Son Tries) or a broad range of herkogamy values (Establiments), consistent with our expectation that approach herkogamy is more common in populations with short-tongued pollinators (Webb and Lloyd 1986; Barrett 2003).

Previous studies on species with continuous variation from reverse to approach herkogamy have shown that the spatial and/or temporal variation in pollinator assemblage could be the mechanism behind the maintenance of such variability. In *P. brandegei*, a species pollinated both by hawkmoths and hummingbirds, hawkmoths selected for reverse herkogamy, whereas hummingbirds selected for approach herkogamy (Kulbaba and Worley 2012, 2013). In *Mertensia fusiformis*, seasonal changes in pollinator-mediated selection were suggested as the factor that maintains the variation in style length (Forrest *et al.* 2011). For differential selection to be the case in *L. implexa*, we needed either a higher preference of a particular pollinator for a type of herkogamy or a higher efficiency of this pollinator when visiting a type of herkogamy, with effects on plant fitness. In our study system, long-tongued pollinators (mostly hawkmoths) preferred to visit plants displaying reverse herkogamy (as in Kulbaba and Worley 2012). Kulbaba and Worley (2012) hypothesized that this preference might be mediated by an effect of exerted floral sex organs on hawkmoth foraging efficiency, since the opening of the corolla tube would be less obstructed; however, the exact mechanism still remains unknown. Differences among populations in visitation patterns, mediated both by pollinator preferences and differences among sites in pollinator assemblages, cascaded into plant reproductive success. In the population where long-tongued pollinators dominate, fruit production was unrelated to herkogamy, but plants with reverse herkogamy sired seeds that germinated better. This pattern suggests a scenario of reduced selfing and increased outcrossing in this population, resulting in moderate pollen transfer across the population (less seeds per fruit, on average) but higher seed quality in outcrossed fruits. In contrast, the two populations with short-tongued insects in the pollinator assemblage showed increased fruit production, but decreased seed quality (seed germination) in flowers with approach herkogamy. This is analogous to the variation in relative fitness of selfed (displaying low separation between anthers and stigma) and outcrossed (with high separation between anthers and stigma) lineages reported for *Datura stramonium* (Motten and Antonovics 1992; Stone and Motten 2002). In flowers with moderate levels of approach herkogamy, the contact of reproductive organs to exerted stigmas probably facilitates pollination by short-tongued visitors, but at the cost of increased selfing, both within plants and within flowers (Fig. 4), and maybe also at the cost of biparental inbreeding, given the low distance that some short-tongued insects, such as beetles, might fly between flowers. The overall higher quality of seeds (seeds that germinated better) from individuals with reverse herkogamy may be related to the fact that long-tongued pollinators are the most effective at transporting outcrossed pollen, as expected in this species with long and thin corolla tubes. This has been found in other species that display also a range from reverse to approach herkogamy (Kulbaba and Worley 2012). Indeed, a previous study conducted in the same populations (Lázaro and Santamaría 2016) supports also this idea, as it showed that where short-tongued insects are important pollinators (Son Tries and Establiments) short corollas were selected for were selected for (see accessibility index), whereas this was not the case in the population where hawkmoths dominated (Banyalbufar).

It is however important to note that we only have studied one population dominated by hawkmoth pollinators. Without replicate populations we cannot completely rule out the possibility that the presence of the long-tongued pollinators and reverse herkogamy are coincidental. Also, we studied the effects on female fitness, thus not including potential effects of conflicting selection among gender functions, which tend to be weak but cannot be disregarded (Sahli and Conner 2011; Kulbaba and Worley 2012). In addition, it might be acknowledged that reproductive assurance may weaken, to some extent, pollinator-mediated selection on floral traits (Lázaro and Totland 2014), that the selective pressures on herkogamy may fluctuate between years as a consequence of changes in pollinator conditions, and that other interactions (e.g. protection from herbivory, nectar robbing and/or mechanical damage by other insects; Parra-Tabla and Bullock 2005; Lázaro and Santamaría 2016) can also modify the selective pressures. Therefore, our study only provides a first depiction of the processes and further studies are required to document the exact mechanisms behind the spatial variation in the relationship between herkogamy and pollinator assemblages. Moreover, the evolutionary importance of selection depends in part on whether variation of the attribute in question has a genetic basis. Although the heritability of continuous variation in style length has been shown in other species (Kulbaba and Worley 2008), studies on the heritability of this trait in *L. implexa* are needed.

## Conclusions

In *L. implexa*, continuous variation from reverse to approach herkogamy might act as a reproductive strategy, optimizing seed quality when long-tongued pollinators are abundant, but increasing fruit set (through self-pollination and pollination by short-tongued insects) when they are rare. As a result, the continuous range of phenotypic variation in herkogamy is compatible with a geographical structured spatial pattern, in which different morphotypes seem to be favoured by the contrasting composition of the local pollinator assemblage.

## Supporting Information

The following additional information is available in the online version of this article—

Table S1. Frequency of visits to plants in total (Arrivals), by different flower-visitor groups (Long- and Short-tongued), and by pollinator orders (Orders) in 15-min observation periods, as well as plants’ number of open flowers (Flowers) and average herkogamy index, at the three study *Lonicera implexa* populations.

plz078_suppl_Supplementary_Material_text

plz078_suppl_Supplementary_Material

plz078_suppl_Supplementary_Material_R

## Data

The data and the R-scripts utilized are available as **Supporting Information**.
