# Supplementary material for: Continuous variation in herkogamy enhances the reproductive response of Lonicera implexa to spatial variation in pollinator assemblages
Source: AoB Plants. 2019 Dec 5;12(1):plz078. doi: 10.1093/aobpla/plz078 (PMC6969618; doi:10.1093/aobpla/plz078)
Supplement: plz078_suppl_Supplementary_Material_R [file plz078_suppl_supplementary_material_r.docx]

**Electronic Supplemental Material- Lázaro et al.**

**Table S1**. Frequency of visits to plants in total (Arrivals), by different flower-visitor groups (Long- and Short-tongued), and by pollinator orders (Orders) in 15 minutes observation periods, as well as plants’ number of open flowers (Flowers) and average Herkogamy index, at the three study *Lonicera implexa* populations.

|  |  |  |  | **Flower visitor** | **groups** |  | **Orders** |  |  | **Herkogamy** |
| --- | --- | --- | --- | --- | --- | --- | --- | --- | --- | --- |
| **Population** | **Plant** | **Flowers** | **Arrivals** | **Long-tongued** | **Short-tongued** | **Coleoptera** | **Diptera** | **Himenoptera** | **Lepidoptera** | **index** |
| Banyalbufar | B1 | 9 | 0 | 0 | 0 | 0 | 0 | 0 | 0 | -2.474 |
| Banyalbufar | B10 | 9 | 0 | 0 | 0 | 0 | 0 | 0 | 0 | -4.965 |
| Banyalbufar | B10 | 9 | 0 | 0 | 0 | 0 | 0 | 0 | 0 | -4.965 |
| Banyalbufar | B10 | 2 | 0 | 0 | 0 | 0 | 0 | 0 | 0 | -4.965 |
| Banyalbufar | B12 | 111 | 2 | 1 | 1 | 1 | 0 | 0 | 1 | -6.581 |
| Banyalbufar | B12 | 45 | 0 | 0 | 0 | 0 | 0 | 0 | 0 | -6.581 |
| Banyalbufar | B12 | 45 | 0 | 0 | 0 | 0 | 0 | 0 | 0 | -6.581 |
| Banyalbufar | B12 | 39 | 0 | 0 | 0 | 0 | 0 | 0 | 0 | -6.581 |
| Banyalbufar | B12 | 6 | 0 | 0 | 0 | 0 | 0 | 0 | 0 | -6.581 |
| Banyalbufar | B12 | 6 | 0 | 0 | 0 | 0 | 0 | 0 | 0 | -6.581 |
| Banyalbufar | B12 | 6 | 0 | 0 | 0 | 0 | 0 | 0 | 0 | -6.581 |
| Banyalbufar | B16 | 896 | 5 | 1 | 4 | 1 | 1 | 2 | 1 | -2.977 |
| Banyalbufar | B16 | 896 | 4 | 1 | 3 | 1 | 0 | 2 | 1 | -2.977 |
| Banyalbufar | B16 | 1422 | 5 | 1 | 4 | 1 | 0 | 3 | 1 | -2.977 |
| Banyalbufar | B16 | 2250 | 4 | 1 | 3 | 1 | 1 | 1 | 1 | -2.977 |
| Banyalbufar | B16 | 186 | 2 | 1 | 1 | 0 | 0 | 1 | 1 | -2.977 |
| Banyalbufar | B16 | 186 | 0 | 0 | 0 | 0 | 0 | 0 | 0 | -2.977 |
| Banyalbufar | B2 | 9 | 0 | 0 | 0 | 0 | 0 | 0 | 0 | 1.145 |
| Banyalbufar | B29 | 3 | 0 | 0 | 0 | 0 | 0 | 0 | 0 | 3.015 |
| Banyalbufar | B32 | 6 | 1 | 1 | 0 | 0 | 0 | 0 | 1 | -3.482 |
| Banyalbufar | B32 | 3 | 0 | 0 | 0 | 0 | 0 | 0 | 0 | -3.482 |
| Banyalbufar | B32 | 3 | 0 | 0 | 0 | 0 | 0 | 0 | 0 | -3.482 |
| Banyalbufar | B35 | 120 | 0 | 0 | 0 | 0 | 0 | 0 | 0 | -4.319 |
| Banyalbufar | B35 | 36 | 0 | 0 | 0 | 0 | 0 | 0 | 0 | -4.319 |
| Banyalbufar | B35 | 36 | 0 | 0 | 0 | 0 | 0 | 0 | 0 | -4.319 |
| Banyalbufar | B36 | 20 | 0 | 0 | 0 | 0 | 0 | 0 | 0 | -0.983 |
| Banyalbufar | B36 | 20 | 0 | 0 | 0 | 0 | 0 | 0 | 0 | -0.983 |
| Banyalbufar | B43 | 45 | 1 | 0 | 1 | 0 | 0 | 1 | 0 | -3.390 |
| Banyalbufar | B43 | 45 | 1 | 0 | 1 | 0 | 0 | 1 | 0 | -3.390 |
| Banyalbufar | B43 | 36 | 1 | 1 | 0 | 0 | 0 | 0 | 1 | -3.390 |
| Banyalbufar | B43 | 36 | 0 | 0 | 0 | 0 | 0 | 0 | 0 | -3.390 |
| Banyalbufar | B43 | 52 | 0 | 0 | 0 | 0 | 0 | 0 | 0 | -3.390 |
| Banyalbufar | B43 | 52 | 0 | 0 | 0 | 0 | 0 | 0 | 0 | -3.390 |
| Banyalbufar | B43 | 52 | 0 | 0 | 0 | 0 | 0 | 0 | 0 | -3.390 |
| Banyalbufar | B44 | 1 | 0 | 0 | 0 | 0 | 0 | 0 | 0 | -1.348 |
| Banyalbufar | B44 | 1 | 0 | 0 | 0 | 0 | 0 | 0 | 0 | -1.348 |
| Banyalbufar | B45 | 102 | 2 | 1 | 1 | 1 | 0 | 0 | 1 | -8.159 |
| Banyalbufar | B45 | 102 | 1 | 1 | 0 | 0 | 0 | 0 | 1 | -8.159 |
| Banyalbufar | B45 | 160 | 1 | 1 | 0 | 0 | 0 | 0 | 1 | -8.159 |
| Banyalbufar | B45 | 48 | 1 | 1 | 0 | 0 | 0 | 0 | 1 | -8.159 |
| Banyalbufar | B45 | 48 | 0 | 0 | 0 | 0 | 0 | 0 | 0 | -8.159 |
| Banyalbufar | B45 | 39 | 1 | 1 | 0 | 0 | 0 | 0 | 1 | -8.159 |
| Banyalbufar | B45 | 39 | 0 | 0 | 0 | 0 | 0 | 0 | 0 | -8.159 |
| Banyalbufar | B46 | 6 | 0 | 0 | 0 | 0 | 0 | 0 | 0 | -4.469 |
| Banyalbufar | B5 | 15 | 0 | 0 | 0 | 0 | 0 | 0 | 0 | -6.844 |
| Banyalbufar | B5 | 3 | 0 | 0 | 0 | 0 | 0 | 0 | 0 | -6.844 |
| Banyalbufar | B5 | 6 | 0 | 0 | 0 | 0 | 0 | 0 | 0 | -6.844 |
| Banyalbufar | B50 | 4 | 0 | 0 | 0 | 0 | 0 | 0 | 0 | 1.575 |
| Banyalbufar | B52 | 6 | 0 | 0 | 0 | 0 | 0 | 0 | 0 | -0.174 |
| Banyalbufar | B52 | 6 | 0 | 0 | 0 | 0 | 0 | 0 | 0 | -0.174 |
| Banyalbufar | B53 | 52 | 0 | 0 | 0 | 0 | 0 | 0 | 0 | -3.238 |
| Banyalbufar | B53 | 52 | 1 | 1 | 0 | 0 | 0 | 0 | 1 | -3.238 |
| Banyalbufar | B53 | 15 | 0 | 0 | 0 | 0 | 0 | 0 | 0 | -3.238 |
| Banyalbufar | B53 | 15 | 0 | 0 | 0 | 0 | 0 | 0 | 0 | -3.238 |
| Banyalbufar | B55 | 4 | 0 | 0 | 0 | 0 | 0 | 0 | 0 | -1.870 |
| Banyalbufar | B55 | 4 | 0 | 0 | 0 | 0 | 0 | 0 | 0 | -1.870 |
| Banyalbufar | B55 | 2 | 0 | 0 | 0 | 0 | 0 | 0 | 0 | -1.870 |
| Banyalbufar | B55 | 2 | 0 | 0 | 0 | 0 | 0 | 0 | 0 | -1.870 |
| Banyalbufar | B6 | 21 | 1 | 0 | 1 | 0 | 1 | 0 | 0 | -0.738 |
| Banyalbufar | B6 | 21 | 0 | 0 | 0 | 0 | 0 | 0 | 0 | -0.738 |
| Banyalbufar | B6 | 3 | 0 | 0 | 0 | 0 | 0 | 0 | 0 | -0.738 |
| Banyalbufar | B6 | 2 | 0 | 0 | 0 | 0 | 0 | 0 | 0 | -0.738 |
| Banyalbufar | B64 | 21 | 0 | 0 | 0 | 0 | 0 | 0 | 0 | -2.778 |
| Banyalbufar | B64 | 12 | 0 | 0 | 0 | 0 | 0 | 0 | 0 | -2.778 |
| Banyalbufar | B64 | 16 | 0 | 0 | 0 | 0 | 0 | 0 | 0 | -2.778 |
| Banyalbufar | B65 | 21 | 0 | 0 | 0 | 0 | 0 | 0 | 0 | -2.315 |
| Banyalbufar | B65 | 2 | 0 | 0 | 0 | 0 | 0 | 0 | 0 | -2.315 |
| Banyalbufar | B65 | 21 | 1 | 0 | 1 | 0 | 1 | 0 | 0 | -2.315 |
| Banyalbufar | B65 | 12 | 0 | 0 | 0 | 0 | 0 | 0 | 0 | -2.315 |
| Banyalbufar | B65 | 12 | 1 | 0 | 1 | 0 | 0 | 1 | 0 | -2.315 |
| Banyalbufar | B65 | 12 | 0 | 0 | 0 | 0 | 0 | 0 | 0 | -2.315 |
| Banyalbufar | B66 | 4 | 0 | 0 | 0 | 0 | 0 | 0 | 0 | -0.412 |
| Banyalbufar | B66 | 27 | 2 | 0 | 2 | 0 | 0 | 2 | 0 | -0.412 |
| Banyalbufar | B66 | 2 | 0 | 0 | 0 | 0 | 0 | 0 | 0 | -0.412 |
| Banyalbufar | B7 | 140 | 1 | 0 | 1 | 1 | 0 | 0 | 0 | -2.702 |
| Banyalbufar | B7 | 140 | 4 | 1 | 3 | 1 | 1 | 1 | 1 | -2.702 |
| Banyalbufar | B7 | 116 | 1 | 1 | 0 | 0 | 0 | 0 | 1 | -2.702 |
| Banyalbufar | B7 | 39 | 0 | 0 | 0 | 0 | 0 | 0 | 0 | -2.702 |
| Banyalbufar | B7 | 9 | 0 | 0 | 0 | 0 | 0 | 0 | 0 | -2.702 |
| Banyalbufar | B76 | 8 | 0 | 0 | 0 | 0 | 0 | 0 | 0 | -2.465 |
| Banyalbufar | B76 | 8 | 0 | 0 | 0 | 0 | 0 | 0 | 0 | -2.465 |
| Banyalbufar | B76 | 6 | 0 | 0 | 0 | 0 | 0 | 0 | 0 | -2.465 |
| Banyalbufar | B76 | 2 | 1 | 1 | 0 | 0 | 0 | 0 | 1 | -2.465 |
| Banyalbufar | B77 | 88 | 0 | 0 | 0 | 0 | 0 | 0 | 0 | -5.407 |
| Banyalbufar | B77 | 8 | 1 | 1 | 0 | 0 | 0 | 0 | 1 | -5.407 |
| Banyalbufar | B77 | 33 | 1 | 1 | 0 | 0 | 0 | 0 | 1 | -5.407 |
| Banyalbufar | B77 | 2 | 0 | 0 | 0 | 0 | 0 | 0 | 0 | -5.407 |
| Banyalbufar | B77 | 2 | 0 | 0 | 0 | 0 | 0 | 0 | 0 | -5.407 |
| Banyalbufar | B77 | 2 | 0 | 0 | 0 | 0 | 0 | 0 | 0 | -5.407 |
| Banyalbufar | B77 | 2 | 0 | 0 | 0 | 0 | 0 | 0 | 0 | -5.407 |
| Banyalbufar | B8 | 12 | 0 | 0 | 0 | 0 | 0 | 0 | 0 | -1.185 |
| Banyalbufar | B8 | 6 | 0 | 0 | 0 | 0 | 0 | 0 | 0 | -1.185 |
| Banyalbufar | B8 | 6 | 0 | 0 | 0 | 0 | 0 | 0 | 0 | -1.185 |
| Banyalbufar | B8 | 9 | 0 | 0 | 0 | 0 | 0 | 0 | 0 | -1.185 |
| Banyalbufar | B8 | 6 | 1 | 0 | 1 | 0 | 1 | 0 | 0 | -1.185 |
| Banyalbufar | B8 | 6 | 0 | 0 | 0 | 0 | 0 | 0 | 0 | -1.185 |
| Banyalbufar | B8 | 6 | 0 | 0 | 0 | 0 | 0 | 0 | 0 | -1.185 |
| Banyalbufar | B9 | 4 | 0 | 0 | 0 | 0 | 0 | 0 | 0 | 2.885 |
| Banyalbufar | B9 | 4 | 0 | 0 | 0 | 0 | 0 | 0 | 0 | 2.885 |
| Banyalbufar | B9 | 1 | 0 | 0 | 0 | 0 | 0 | 0 | 0 | 2.885 |
| Banyalbufar | B9 | 1 | 0 | 0 | 0 | 0 | 0 | 0 | 0 | 2.885 |
| Establiments | E1 | 20 | 0 | 0 | 0 | 0 | 0 | 0 | 0 | -1.091 |
| Establiments | E1 | 128 | 1 | 0 | 1 | 1 | 0 | 0 | 0 | -1.091 |
| Establiments | E13 | 64 | 1 | 0 | 1 | 1 | 0 | 0 | 0 | 2.944 |
| Establiments | E14 | 12 | 1 | 0 | 1 | 1 | 0 | 0 | 0 | -3.702 |
| Establiments | E15 | 24 | 1 | 0 | 1 | 1 | 0 | 0 | 0 | -2.798 |
| Establiments | E15 | 24 | 1 | 0 | 1 | 1 | 0 | 0 | 0 | -2.798 |
| Establiments | E2 | 48 | 0 | 0 | 0 | 0 | 0 | 0 | 0 | 2.827 |
| Establiments | E2 | 84 | 0 | 0 | 0 | 0 | 0 | 0 | 0 | 2.827 |
| Establiments | E20 | 216 | 2 | 0 | 2 | 1 | 0 | 1 | 0 | 2.154 |
| Establiments | E20 | 332 | 5 | 0 | 5 | 1 | 1 | 3 | 0 | 2.154 |
| Establiments | E20 | 8 | 0 | 0 | 0 | 0 | 0 | 0 | 0 | 2.154 |
| Establiments | E21 | 136 | 3 | 0 | 3 | 2 | 0 | 1 | 0 | 2.906 |
| Establiments | E21 | 96 | 1 | 0 | 1 | 1 | 0 | 0 | 0 | 2.906 |
| Establiments | E21 | 80 | 2 | 0 | 2 | 2 | 0 | 0 | 0 | 2.906 |
| Establiments | E21 | 6 | 0 | 0 | 0 | 0 | 0 | 0 | 0 | 2.906 |
| Establiments | E22 | 40 | 3 | 0 | 3 | 2 | 0 | 1 | 0 | -4.206 |
| Establiments | E22 | 32 | 0 | 0 | 0 | 0 | 0 | 0 | 0 | -4.206 |
| Establiments | E22 | 48 | 1 | 0 | 1 | 1 | 0 | 0 | 0 | -4.206 |
| Establiments | E22 | 1 | 0 | 0 | 0 | 0 | 0 | 0 | 0 | -4.206 |
| Establiments | E23 | 84 | 1 | 0 | 1 | 1 | 0 | 0 | 0 | -1.402 |
| Establiments | E23 | 84 | 0 | 0 | 0 | 0 | 0 | 0 | 0 | -1.402 |
| Establiments | E23 | 12 | 0 | 0 | 0 | 0 | 0 | 0 | 0 | -1.402 |
| Establiments | E24 | 40 | 0 | 0 | 0 | 0 | 0 | 0 | 0 | -2.936 |
| Establiments | E24 | 56 | 2 | 0 | 2 | 1 | 1 | 0 | 0 | -2.936 |
| Establiments | E24 | 90 | 1 | 0 | 1 | 0 | 1 | 0 | 0 | -2.936 |
| Establiments | E24 | 21 | 1 | 0 | 1 | 0 | 1 | 0 | 0 | -2.936 |
| Establiments | E25 | 40 | 0 | 0 | 0 | 0 | 0 | 0 | 0 | -4.116 |
| Establiments | E25 | 108 | 1 | 0 | 1 | 1 | 0 | 0 | 0 | -4.116 |
| Establiments | E25 | 115 | 2 | 0 | 2 | 1 | 1 | 0 | 0 | -4.116 |
| Establiments | E25 | 28 | 0 | 0 | 0 | 0 | 0 | 0 | 0 | -4.116 |
| Establiments | E29 | 152 | 0 | 0 | 0 | 0 | 0 | 0 | 0 | -5.485 |
| Establiments | E29 | 60 | 2 | 0 | 2 | 2 | 0 | 0 | 0 | -5.485 |
| Establiments | E29 | 176 | 0 | 0 | 0 | 0 | 0 | 0 | 0 | -5.485 |
| Establiments | E29 | 51 | 0 | 0 | 0 | 0 | 0 | 0 | 0 | -5.485 |
| Establiments | E30 | 88 | 0 | 0 | 0 | 0 | 0 | 0 | 0 | 4.550 |
| Establiments | E30 | 80 | 3 | 0 | 3 | 1 | 2 | 0 | 0 | 4.550 |
| Establiments | E30 | 4 | 0 | 0 | 0 | 0 | 0 | 0 | 0 | 4.550 |
| Establiments | E31 | 24 | 0 | 0 | 0 | 0 | 0 | 0 | 0 | -0.306 |
| Establiments | E31 | 12 | 0 | 0 | 0 | 0 | 0 | 0 | 0 | -0.306 |
| Establiments | E31 | 4 | 0 | 0 | 0 | 0 | 0 | 0 | 0 | -0.306 |
| Establiments | E32 | 288 | 2 | 0 | 2 | 1 | 0 | 1 | 0 | 3.170 |
| Establiments | E32 | 1536 | 2 | 0 | 2 | 2 | 0 | 0 | 0 | 3.170 |
| Establiments | E32 | 165 | 4 | 0 | 4 | 2 | 0 | 2 | 0 | 3.170 |
| Establiments | E32 | 152 | 1 | 0 | 1 | 1 | 0 | 0 | 0 | 3.170 |
| Establiments | E32 | 8 | 0 | 0 | 0 | 0 | 0 | 0 | 0 | 3.170 |
| Establiments | E37 | 20 | 0 | 0 | 0 | 0 | 0 | 0 | 0 | -2.965 |
| Establiments | E37 | 20 | 1 | 0 | 1 | 1 | 0 | 0 | 0 | -2.965 |
| Establiments | E37 | 12 | 0 | 0 | 0 | 0 | 0 | 0 | 0 | -2.965 |
| Establiments | E37 | 6 | 0 | 0 | 0 | 0 | 0 | 0 | 0 | -2.965 |
| Establiments | E38 | 48 | 0 | 0 | 0 | 0 | 0 | 0 | 0 | -5.170 |
| Establiments | E38 | 100 | 1 | 0 | 1 | 1 | 0 | 0 | 0 | -5.170 |
| Establiments | E38 | 87 | 0 | 0 | 0 | 0 | 0 | 0 | 0 | -5.170 |
| Establiments | E38 | 92 | 0 | 0 | 0 | 0 | 0 | 0 | 0 | -5.170 |
| Establiments | E39 | 112 | 1 | 0 | 1 | 1 | 0 | 0 | 0 | 1.231 |
| Establiments | E39 | 168 | 0 | 0 | 0 | 0 | 0 | 0 | 0 | 1.231 |
| Establiments | E39 | 90 | 0 | 0 | 0 | 0 | 0 | 0 | 0 | 1.231 |
| Establiments | E39 | 10 | 0 | 0 | 0 | 0 | 0 | 0 | 0 | 1.231 |
| Establiments | E4 | 1168 | 3 | 1 | 2 | 1 | 0 | 1 | 1 | -1.391 |
| Establiments | E40 | 16 | 1 | 0 | 1 | 1 | 0 | 0 | 0 | 1.752 |
| Establiments | E40 | 12 | 1 | 0 | 1 | 1 | 0 | 0 | 0 | 1.752 |
| Establiments | E40 | 12 | 0 | 0 | 0 | 0 | 0 | 0 | 0 | 1.752 |
| Establiments | E42 | 16 | 0 | 0 | 0 | 0 | 0 | 0 | 0 | -1.010 |
| Establiments | E44 | 56 | 2 | 0 | 2 | 2 | 0 | 0 | 0 | 0.282 |
| Establiments | E44 | 76 | 0 | 0 | 0 | 0 | 0 | 0 | 0 | 0.282 |
| Establiments | E44 | 40 | 0 | 0 | 0 | 0 | 0 | 0 | 0 | 0.282 |
| Establiments | E44 | 12 | 0 | 0 | 0 | 0 | 0 | 0 | 0 | 0.282 |
| Establiments | E45 | 128 | 2 | 0 | 2 | 2 | 0 | 0 | 0 | 1.665 |
| Establiments | E45 | 128 | 1 | 0 | 1 | 0 | 0 | 1 | 0 | 1.665 |
| Establiments | E45 | 40 | 0 | 0 | 0 | 0 | 0 | 0 | 0 | 1.665 |
| Establiments | E45 | 48 | 0 | 0 | 0 | 0 | 0 | 0 | 0 | 1.665 |
| Establiments | E46 | 100 | 1 | 0 | 1 | 1 | 0 | 0 | 0 | -4.263 |
| Establiments | E46 | 92 | 1 | 0 | 1 | 1 | 0 | 0 | 0 | -4.263 |
| Establiments | E46 | 95 | 0 | 0 | 0 | 0 | 0 | 0 | 0 | -4.263 |
| Establiments | E46 | 56 | 0 | 0 | 0 | 0 | 0 | 0 | 0 | -4.263 |
| Establiments | E47 | 20 | 0 | 0 | 0 | 0 | 0 | 0 | 0 | 0.671 |
| Establiments | E47 | 30 | 2 | 0 | 2 | 2 | 0 | 0 | 0 | 0.671 |
| Establiments | E47 | 20 | 1 | 0 | 1 | 1 | 0 | 0 | 0 | 0.671 |
| Establiments | E47 | 24 | 0 | 0 | 0 | 0 | 0 | 0 | 0 | 0.671 |
| Establiments | E47bis | 108 | 2 | 0 | 2 | 2 | 0 | 0 | 0 | -1.208 |
| Establiments | E47bis | 92 | 1 | 0 | 1 | 1 | 0 | 0 | 0 | -1.208 |
| Establiments | E47bis | 280 | 2 | 0 | 2 | 2 | 0 | 0 | 0 | -1.208 |
| Establiments | E47bis | 24 | 0 | 0 | 0 | 0 | 0 | 0 | 0 | -1.208 |
| Establiments | E48 | 152 | 1 | 0 | 1 | 1 | 0 | 0 | 0 | 1.077 |
| Establiments | E48 | 120 | 2 | 0 | 2 | 2 | 0 | 0 | 0 | 1.077 |
| Establiments | E48 | 68 | 0 | 0 | 0 | 0 | 0 | 0 | 0 | 1.077 |
| Establiments | E49 | 24 | 0 | 0 | 0 | 0 | 0 | 0 | 0 | 5.210 |
| Establiments | E49 | 104 | 2 | 0 | 1 | 1 | 0 | 0 | 0 | 5.210 |
| Establiments | E49 | 130 | 1 | 0 | 1 | 1 | 0 | 0 | 0 | 5.210 |
| Establiments | E49 | 39 | 1 | 0 | 1 | 1 | 0 | 0 | 0 | 5.210 |
| Establiments | E51 | 128 | 0 | 0 | 0 | 0 | 0 | 0 | 0 | -0.636 |
| Establiments | E51 | 88 | 1 | 0 | 1 | 1 | 0 | 0 | 0 | -0.636 |
| Establiments | E51 | 32 | 1 | 0 | 1 | 1 | 0 | 0 | 0 | -0.636 |
| Establiments | E52 | 64 | 0 | 0 | 0 | 0 | 0 | 0 | 0 | 5.139 |
| Establiments | E52 | 196 | 2 | 0 | 2 | 2 | 0 | 0 | 0 | 5.139 |
| Establiments | E52 | 395 | 0 | 0 | 0 | 0 | 0 | 0 | 0 | 5.139 |
| Establiments | E52 | 304 | 2 | 1 | 1 | 0 | 0 | 1 | 1 | 5.139 |
| Establiments | E53 | 84 | 0 | 0 | 0 | 0 | 0 | 0 | 0 | -2.236 |
| Establiments | E53 | 124 | 1 | 0 | 1 | 0 | 0 | 1 | 0 | -2.236 |
| Establiments | E53 | 21 | 0 | 0 | 0 | 0 | 0 | 0 | 0 | -2.236 |
| Establiments | E54 | 104 | 0 | 0 | 0 | 0 | 0 | 0 | 0 | -1.928 |
| Establiments | E54 | 72 | 0 | 0 | 0 | 0 | 0 | 0 | 0 | -1.928 |
| Establiments | E54 | 4 | 0 | 0 | 0 | 0 | 0 | 0 | 0 | -1.928 |
| Establiments | E55 | 148 | 1 | 0 | 1 | 0 | 1 | 0 | 0 | 3.699 |
| Establiments | E55 | 212 | 0 | 0 | 0 | 0 | 0 | 0 | 0 | 3.699 |
| Establiments | E55 | 24 | 0 | 0 | 0 | 0 | 0 | 0 | 0 | 3.699 |
| Establiments | E56 | 28 | 0 | 0 | 0 | 0 | 0 | 0 | 0 | -0.094 |
| Establiments | E56 | 76 | 0 | 0 | 0 | 0 | 0 | 0 | 0 | -0.094 |
| Establiments | E6 | 16 | 1 | 0 | 1 | 1 | 0 | 0 | 0 | -4.103 |
| Establiments | E7 | 44 | 0 | 0 | 0 | 0 | 0 | 0 | 0 | -6.306 |
| Son Tries | ST1 | 200 | 0 | 0 | 0 | 0 | 0 | 0 | 0 | -5.800 |
| Son Tries | ST1 | 21 | 1 | 1 | 0 | 0 | 0 | 0 | 1 | -5.800 |
| Son Tries | ST1 | 6 | 0 | 0 | 0 | 0 | 0 | 0 | 0 | -5.800 |
| Son Tries | ST10 | 220 | 3 | 0 | 3 | 3 | 0 | 0 | 0 | 0.524 |
| Son Tries | ST10 | 220 | 2 | 0 | 2 | 1 | 0 | 1 | 0 | 0.524 |
| Son Tries | ST12 | 21 | 0 | 0 | 0 | 0 | 0 | 0 | 0 | 1.275 |
| Son Tries | ST12 | 4 | 0 | 0 | 0 | 0 | 0 | 0 | 0 | 1.275 |
| Son Tries | ST13 | 51 | 1 | 0 | 1 | 0 | 1 | 0 | 0 | 3.907 |
| Son Tries | ST13 | 32 | 0 | 0 | 0 | 0 | 0 | 0 | 0 | 3.907 |
| Son Tries | ST13 | 8 | 0 | 0 | 0 | 0 | 0 | 0 | 0 | 3.907 |
| Son Tries | ST15 | 648 | 3 | 1 | 2 | 1 | 1 | 0 | 1 | 1.440 |
| Son Tries | ST15 | 18 | 1 | 0 | 1 | 0 | 0 | 1 | 0 | 1.440 |
| Son Tries | ST15 | 339 | 0 | 0 | 0 | 0 | 0 | 0 | 0 | 1.440 |
| Son Tries | ST17 | 102 | 1 | 0 | 1 | 1 | 0 | 0 | 0 | 2.392 |
| Son Tries | ST17 | 102 | 2 | 0 | 2 | 1 | 0 | 1 | 0 | 2.392 |
| Son Tries | ST17 | 8 | 0 | 0 | 0 | 0 | 0 | 0 | 0 | 2.392 |
| Son Tries | ST18 | 270 | 2 | 0 | 2 | 1 | 0 | 1 | 0 | 0.166 |
| Son Tries | ST18 | 24 | 1 | 0 | 1 | 1 | 0 | 0 | 0 | 0.166 |
| Son Tries | ST18bis | 40 | 1 | 0 | 1 | 1 | 0 | 0 | 0 | 3.630 |
| Son Tries | ST18bis | 15 | 1 | 0 | 1 | 1 | 0 | 0 | 0 | 3.630 |
| Son Tries | ST19 | 340 | 3 | 1 | 2 | 1 | 0 | 1 | 1 | 0.027 |
| Son Tries | ST19 | 104 | 2 | 0 | 2 | 1 | 0 | 1 | 0 | 0.027 |
| Son Tries | ST19 | 12 | 0 | 0 | 0 | 0 | 0 | 0 | 0 | 0.027 |
| Son Tries | ST21 | 52 | 0 | 0 | 0 | 0 | 0 | 0 | 0 | 1.564 |
| Son Tries | ST21 | 9 | 0 | 0 | 0 | 0 | 0 | 0 | 0 | 1.564 |
| Son Tries | ST21 | 3 | 0 | 0 | 0 | 0 | 0 | 0 | 0 | 1.564 |
| Son Tries | ST22 | 140 | 1 | 0 | 1 | 0 | 1 | 0 | 0 | 2.113 |
| Son Tries | ST22 | 528 | 2 | 1 | 1 | 1 | 0 | 0 | 1 | 2.113 |
| Son Tries | ST22 | 30 | 0 | 0 | 0 | 0 | 0 | 0 | 0 | 2.113 |
| Son Tries | ST23 | 144 | 2 | 0 | 2 | 1 | 1 | 0 | 0 | 2.888 |
| Son Tries | ST24 | 16 | 1 | 0 | 1 | 1 | 0 | 0 | 0 | 0.013 |
| Son Tries | ST25 | 68 | 2 | 2 | 0 | 0 | 0 | 0 | 2 | 0.272 |
| Son Tries | ST25 | 6 | 0 | 0 | 0 | 0 | 0 | 0 | 0 | 0.272 |
| Son Tries | ST26 | 78 | 0 | 0 | 0 | 0 | 0 | 0 | 0 | 0.977 |
| Son Tries | ST26 | 280 | 3 | 1 | 2 | 1 | 1 | 0 | 1 | 0.977 |
| Son Tries | ST26 | 6 | 0 | 0 | 0 | 0 | 0 | 0 | 0 | 0.977 |
| Son Tries | ST27 | 20 | 0 | 0 | 0 | 0 | 0 | 0 | 0 | 3.362 |
| Son Tries | ST27 | 12 | 0 | 0 | 0 | 0 | 0 | 0 | 0 | 3.362 |
| Son Tries | ST27 | 2 | 0 | 0 | 0 | 0 | 0 | 0 | 0 | 3.362 |
| Son Tries | ST3 | 18 | 0 | 0 | 0 | 0 | 0 | 0 | 0 | -0.361 |
| Son Tries | ST3 | 12 | 0 | 0 | 0 | 0 | 0 | 0 | 0 | -0.361 |
| Son Tries | ST3 | 6 | 0 | 0 | 0 | 0 | 0 | 0 | 0 | -0.361 |
| Son Tries | ST30 | 6 | 0 | 0 | 0 | 0 | 0 | 0 | 0 | 1.005 |
| Son Tries | ST30 | 3 | 0 | 0 | 0 | 0 | 0 | 0 | 0 | 1.005 |
| Son Tries | ST30 | 3 | 0 | 0 | 0 | 0 | 0 | 0 | 0 | 1.005 |
| Son Tries | ST31 | 21 | 3 | 0 | 3 | 2 | 0 | 1 | 0 | -0.438 |
| Son Tries | ST31 | 27 | 1 | 1 | 0 | 0 | 0 | 0 | 1 | -0.438 |
| Son Tries | ST31 | 28 | 1 | 0 | 1 | 1 | 0 | 0 | 0 | -0.438 |
| Son Tries | ST31 | 2 | 0 | 0 | 0 | 0 | 0 | 0 | 0 | -0.438 |
| Son Tries | ST32 | 12 | 1 | 0 | 1 | 1 | 0 | 0 | 0 | -3.987 |
| Son Tries | ST32 | 15 | 0 | 0 | 0 | 0 | 0 | 0 | 0 | -3.987 |
| Son Tries | ST32 | 8 | 1 | 0 | 1 | 1 | 0 | 0 | 0 | -3.987 |
| Son Tries | ST32 | 2 | 0 | 0 | 0 | 0 | 0 | 0 | 0 | -3.987 |
| Son Tries | ST35 | 224 | 1 | 0 | 1 | 0 | 0 | 1 | 0 | 0.026 |
| Son Tries | ST35 | 176 | 4 | 2 | 2 | 2 | 0 | 0 | 2 | 0.026 |
| Son Tries | ST35 | 176 | 3 | 0 | 3 | 2 | 0 | 1 | 0 | 0.026 |
| Son Tries | ST35 | 104 | 1 | 1 | 0 | 0 | 0 | 0 | 1 | 0.026 |
| Son Tries | ST35 | 36 | 0 | 0 | 0 | 0 | 0 | 0 | 0 | 0.026 |
| Son Tries | ST36 | 408 | 4 | 0 | 4 | 1 | 1 | 2 | 0 | -1.105 |
| Son Tries | ST36 | 408 | 2 | 0 | 2 | 1 | 0 | 1 | 0 | -1.105 |
| Son Tries | ST36 | 4 | 0 | 0 | 0 | 0 | 0 | 0 | 0 | -1.105 |
| Son Tries | ST36 | 6 | 0 | 0 | 0 | 0 | 0 | 0 | 0 | -1.105 |
| Son Tries | ST37 | 15 | 0 | 0 | 0 | 0 | 0 | 0 | 0 | 0.693 |
| Son Tries | ST37 | 3 | 0 | 0 | 0 | 0 | 0 | 0 | 0 | 0.693 |
| Son Tries | ST37 | 2 | 0 | 0 | 0 | 0 | 0 | 0 | 0 | 0.693 |
| Son Tries | ST38 | 68 | 0 | 0 | 0 | 0 | 0 | 0 | 0 | -1.624 |
| Son Tries | ST38 | 68 | 1 | 1 | 0 | 0 | 0 | 0 | 1 | -1.624 |
| Son Tries | ST38 | 20 | 0 | 0 | 0 | 0 | 0 | 0 | 0 | -1.624 |
| Son Tries | ST38 | 4 | 0 | 0 | 0 | 0 | 0 | 0 | 0 | -1.624 |
| Son Tries | ST4 | 220 | 3 | 0 | 3 | 1 | 1 | 1 | 0 | 2.282 |
| Son Tries | ST4 | 220 | 1 | 0 | 1 | 0 | 0 | 1 | 0 | 2.282 |
| Son Tries | ST4 | 320 | 1 | 0 | 1 | 0 | 0 | 1 | 0 | 2.282 |
| Son Tries | ST4 | 66 | 2 | 1 | 1 | 1 | 0 | 0 | 1 | 2.282 |
| Son Tries | ST40 | 20 | 1 | 0 | 1 | 0 | 0 | 1 | 0 | -1.384 |
| Son Tries | ST40 | 12 | 0 | 0 | 0 | 0 | 0 | 0 | 0 | -1.384 |
| Son Tries | ST40 | 1 | 0 | 0 | 0 | 0 | 0 | 0 | 0 | -1.384 |
| Son Tries | ST40bis | 9 | 1 | 0 | 1 | 0 | 1 | 0 | 0 | 0.197 |
| Son Tries | ST40bis | 3 | 0 | 0 | 0 | 0 | 0 | 0 | 0 | 0.197 |
| Son Tries | ST40bis | 2 | 0 | 0 | 0 | 0 | 0 | 0 | 0 | 0.197 |
| Son Tries | ST41 | 256 | 2 | 1 | 1 | 1 | 0 | 0 | 1 | 0.626 |
| Son Tries | ST41 | 256 | 2 | 1 | 1 | 1 | 0 | 0 | 1 | 0.626 |
| Son Tries | ST41 | 84 | 3 | 0 | 3 | 2 | 0 | 1 | 0 | 0.626 |
| Son Tries | ST41 | 8 | 0 | 0 | 0 | 0 | 0 | 0 | 0 | 0.626 |
| Son Tries | ST42 | 56 | 3 | 0 | 3 | 2 | 1 | 0 | 0 | 3.825 |
| Son Tries | ST42 | 114 | 2 | 1 | 1 | 1 | 0 | 0 | 1 | 3.825 |
| Son Tries | ST42 | 102 | 0 | 0 | 0 | 0 | 0 | 0 | 0 | 3.825 |
| Son Tries | ST44 | 40 | 2 | 0 | 2 | 1 | 1 | 0 | 0 | 2.124 |
| Son Tries | ST44 | 4 | 0 | 0 | 0 | 0 | 0 | 0 | 0 | 2.124 |
| Son Tries | ST44 | 2 | 0 | 0 | 0 | 0 | 0 | 0 | 0 | 2.124 |
| Son Tries | ST47 | 24 | 1 | 0 | 1 | 0 | 1 | 0 | 0 | 2.387 |
| Son Tries | ST47 | 2 | 1 | 0 | 1 | 1 | 0 | 0 | 0 | 2.387 |
| Son Tries | ST50 | 144 | 2 | 1 | 1 | 1 | 0 | 0 | 1 | -2.615 |
| Son Tries | ST50 | 144 | 2 | 1 | 1 | 1 | 0 | 0 | 1 | -2.615 |
| Son Tries | ST50 | 136 | 3 | 1 | 2 | 1 | 0 | 1 | 1 | -2.615 |
| Son Tries | ST50 | 136 | 2 | 1 | 1 | 1 | 0 | 0 | 1 | -2.615 |
| Son Tries | ST50 | 27 | 0 | 0 | 0 | 0 | 0 | 0 | 0 | -2.615 |
| Son Tries | ST53 | 20 | 1 | 0 | 1 | 1 | 0 | 0 | 0 | -1.169 |
| Son Tries | ST53 | 4 | 0 | 0 | 0 | 0 | 0 | 0 | 0 | -1.169 |
| Son Tries | ST53 | 3 | 0 | 0 | 0 | 0 | 0 | 0 | 0 | -1.169 |
